# A multi-center, prospective cohort study of whole blood gene expression in the tuberculosis-diabetes interaction

**DOI:** 10.1038/s41598-023-34847-9

**Published:** 2023-05-12

**Authors:** Artur T. L. Queiroz, Caian L. Vinhaes, Eduardo R. Fukutani, Akshay N. Gupte, Nathella Pavan Kumar, Kiyoshi F. Fukutani, María B. Arriaga, Timothy R. Sterling, Subash Babu, Sanjay Gaikwad, Rajesh Karyakarte, Vidya Mave, Mandar Paradhkar, Vijay Viswanathan, Amita Gupta, Bruno B. Andrade, Hardy Kornfeld, the RePORT Brazil, Alice M. S. Andrade, Marina C. Figueiredo, Vanessa Nascimento, Juan Manuel Cubillos-Angulo, Hayna Malta-Santos, Jéssica Rebouças-Silva, Adriano Gomes-Silva, Saulo R. N. Santos, André Ramos, Pedro Brito, Carolina A. S. Schmaltz, Alysson G. Costa, Leandro Sousa Garcia, Brenda K. de Sousa Carvalho, Bruna P. de Loiola, Francine P. Ignácio, Maria C. Lourenço, Elisangela C. Silva, Mayla Mello, Alexandra B. Souza, Michael S. Rocha, Aline Benjamin, Adriana S. R. Moreira, Jamile G. de Oliveira, Solange Cavalcante, Betina Durovni, Marcelo Cordeiro-Santos, Afrânio L. Kristki, Valeria C. Rolla, José R. Lapa-e-Silva, RePORT India Consortia, Kim West, Vandana Kulkami, Nikhil Gupte

**Affiliations:** 1 Centro de Integração de Dados e Conhecimentos para Saúde, Instituto Gonçalo Moniz, Fundação Oswaldo Cruz, Salvador, Brazil; 2 Laboratório de Inflamação e Biomarcadores, Instituto Gonçalo Moniz, Fundação Oswaldo Cruz, Salvador, Brazil; 3 Multinational Organization Network Sponsoring Translational and Epidemiological Research (MONSTER) Initiative, Salvador, 41810‑710 Brazil; 4 Escola Bahiana de Medicina e Saúde Pública (EBMSP), Salvador, 40290-150 Brazil; 5 Johns Hopkins Bloomberg School of Public Health, Baltimore, MD USA; 6 National Institutes of Health‐ NIRT ‐ International Center for Excellence in Research, Chennai, India; 7 Division of Infectious Diseases, Department of Medicine, Vanderbilt University Medical Center, Nashville, TN USA; 8 Department of Pulmonary Medicine, Byramjee-Jeejeebhoy Government Medical College and Sassoon General Hospitals, Pune, India; 9 Department of Microbiology, Byramjee-Jeejeebhoy Government Medical College and Sassoon General Hospitals, Pune, India; 10 Byramjee-Jeejeebhoy Government Medical College-Johns Hopkins University Clinical Research Site, Pune, India; 11 Johns Hopkins Center for Infectious Diseases in India, Pune, India; 12 Prof. M. Viswanathan Diabetes Research Centre, Chennai, India; 13 Faculdade de Tecnologia e Ciências, Instituto de Pesquisa Clínica e Translacional, Salvador, 41741-590 Brazil; 14 Department of Medicine, University of Massachusetts Medical School, Worcester, MA USA; 15 UMass Chan Medical School, Worcester, MA USA; 16 Instituto Brasileiro para Investigação da Tuberculose, Fundação José Silveira, Salvador, Brazil; 17 Faculdade de Medicina, Universidade Federal da Bahia, Salvador, Brazil; 18 Laboratório de Pesquisa Clínica em Micobacteriose, Instituto Nacional de Infectologia Evandro Chagas, Fiocruz, Rio de Janeiro, Brazil; 19 Fundação Medicina Tropical Dr Heitor Vieira Dourado, Manaus, Brazil; 20 Programa de Pós-Graduação em Medicina Tropical, Universidade do Estado do Amazonas, Manaus, Brazil; 21 Universidade Federal do Amazonas, Manaus, Brazil; 22 Programa Acadêmico de Tuberculose da Faculdade de Medicina, Universidade Federal do Rio de Janeiro, Rio de Janeiro, Brazil; 23 Instituto Nacional de Infectologia Evandro Chagas, Fiocruz, Rio de Janeiro, Brazil; 24 Faculdade de Medicina, Programa Acadêmico de Tuberculose, Universidade Federal do Rio de Janeiro, Rio de Janeiro, Brazil

**Keywords:** Infection, Infectious diseases, Inflammation, Biomarkers, Medical research, Molecular medicine

## Abstract

Diabetes mellitus (DM) increases tuberculosis (TB) severity. We compared blood gene expression in adults with pulmonary TB, with or without diabetes mellitus (DM) from sites in Brazil and India. RNA sequencing (RNAseq) performed at baseline and during TB treatment. Publicly available baseline RNAseq data from South Africa and Romania reported by the TANDEM Consortium were also analyzed. Across the sites, differentially expressed genes varied for each condition (DM, TB, and TBDM) and no pattern classified any one group across all sites. A concise signature of TB disease was identified but this was expressed equally in TB and TBDM. Pathway enrichment analysis failed to distinguish TB from TBDM, although there was a trend for greater neutrophil and innate immune pathway activation in TBDM participants. Pathways associated with insulin resistance, metabolic dysfunction, diabetic complications, and chromosomal instability were positively correlated with glycohemoglobin. The immune response to pulmonary TB as reflected by whole blood gene expression is substantially similar with or without comorbid DM. Gene expression pathways associated with the microvascular and macrovascular complications of DM are upregulated during TB, supporting a syndemic interaction between these coprevalent diseases.

## Introduction

Diabetes mellitus (DM) has been associated with increased risk for tuberculosis (TB) progression and adverse TB treatment outcomes in most clinical studies^1^. Mirroring the human data, animal models combining chronic hyperglycemia with *Mycobacterium tuberculosis* challenge showed higher lung bacterial burden and more TB immune pathology^2^. The global population-attributable fraction of TB associated with DM is comparable to that of HIV/AIDS^3^. Despite its significance as a barrier to TB elimination^4^, the mechanisms whereby DM impairs host defense against *M*. *tuberculosis* are not well understood^5^.

Mechanistic studies of human immunity to TB are limited to accessible tissue samples. Blood transcriptomic studies have described a consensus TB signature of increased type I interferon (IFN) signaling^6^. In a prior blood transcriptome study using microarrays to evaluate a South Indian cohort, we found no pattern of immune gene expression that distinguished TB-DM comorbidity from TB in participants without DM^7^. The TANDEM Consortium later published a whole blood RNA sequencing (RNAseq) study from four national sites (South Africa, Peru, Indonesia, Romania)^8^. Their analysis revealed increased inflammatory pathway gene expression but reduced type 1 interferon signaling in TB combined with dysglycemia compared to TB in people with normoglycemia. To further explore the impact of DM on the host response to TB in diverse populations, we performed RNAseq on whole blood RNA sampled at TB diagnosis (baseline) and treatment months 2 and 6 from adults with pulmonary TB, with or without DM, at sites in India and Brazil.

The Molecular Signatures of Tuberculosis-Diabetes Interaction (MSTDI) study reported here leveraged unreported participant data and whole blood RNA samples from prospective observational pulmonary TB cohorts from two sites of the RePORT India and one from RePORT Brazil consortia. A single vendor performed RNAseq on baseline samples from all three sites and longitudinal samples during TB treatment from one Indian site and Brazil. To check the variability of the blood transcriptome in TB-DM interaction, comparison was made with published blood RNAseq data from two TANDEM consortium sites (Romania and South Africa). Our analysis was aimed to compare the intensity and quality of inflammatory activation between the clinical conditions and sites and evaluate the impact of HbA1c levels in the biological pathways. the This investigation did not reveal insights to the mechanisms of TB susceptibility in DM but the data support the existence of a syndemic interaction between TB and DM^9^.

## Results

### Differential gene expression between clinical conditions and sites

The MSTDI cohort, comprising 290 participants, was recruited from two sites in India and one site in Brazil. Adults newly diagnosed with drug-sensitive pulmonary TB, with or without DM (TB and TBDM, respectively), and control group participants without TB, with or without DM (DM and HC, respectively) were enrolled. Characteristics of the population are shown in Supplementary Fig. S1 and Supplementary Tables S1 and S2. Additional comparison was made with publicly available RNAseq data from two sites (Romania and South Africa) of a TANDEM consortium gene expression study with similar group structure and where data were available for site-specific healthy control (HC) participants.

**Figure 1:**
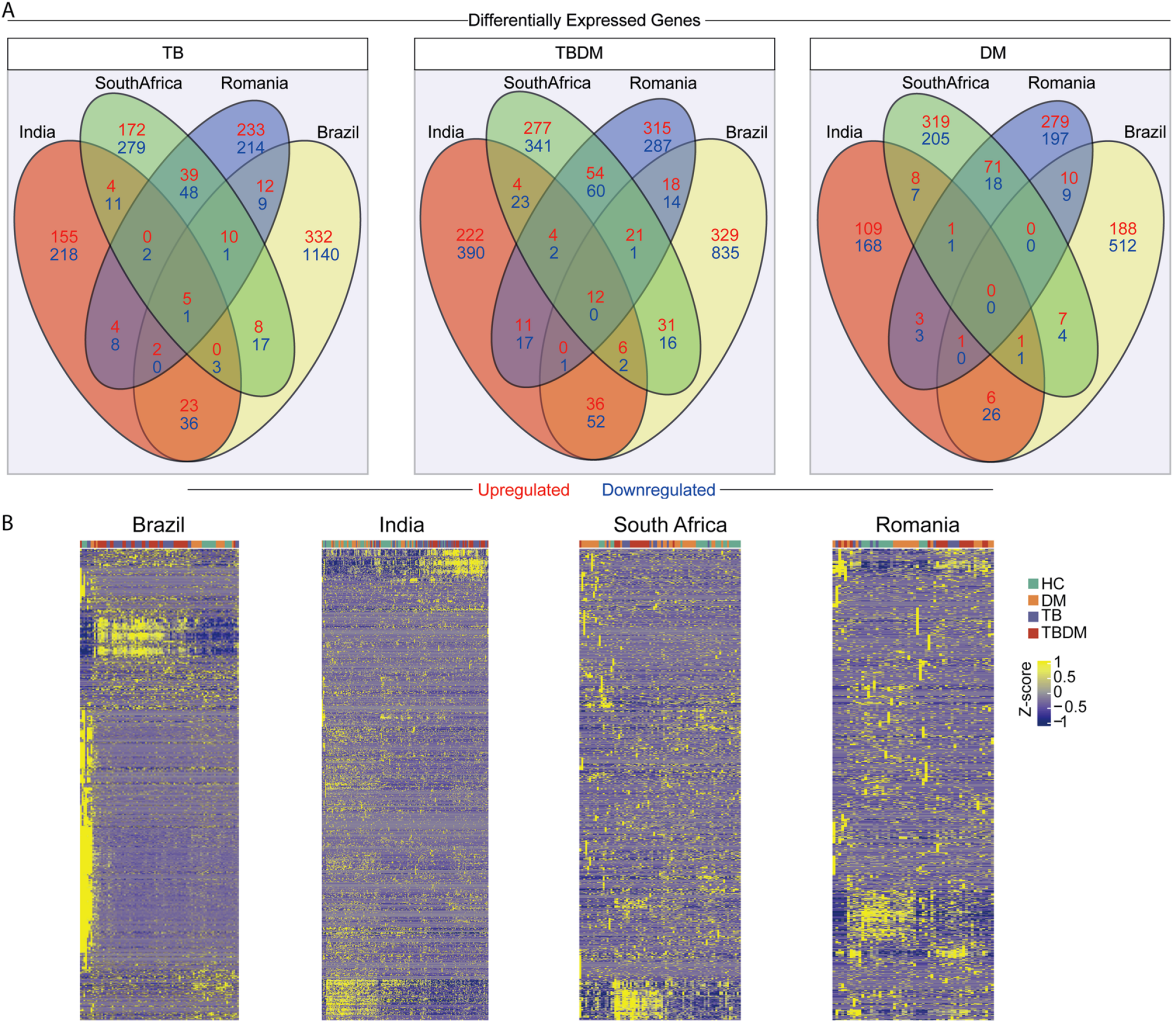
Distribution of differentially expressed genes (DEGs) between clinical groups in the sites from MSTDI and TANDEM cohorts. (**A**) Venn Diagrams show the DEGs defined by the threshold corrected p-value < 0.05 and log twofold change >  + /– 1.4 from each comparison of TB vs, TBDM vs, and DM vs HC from the subjects within the cohort sites. (**B**) Heat maps of z-score normalized data of all DEGs from each cohort site. Each heat map depicts all DEGS from TB vs, TBDM vs, and DM vs HC comparison from subjects within each cohort site.

**Figure 2:**
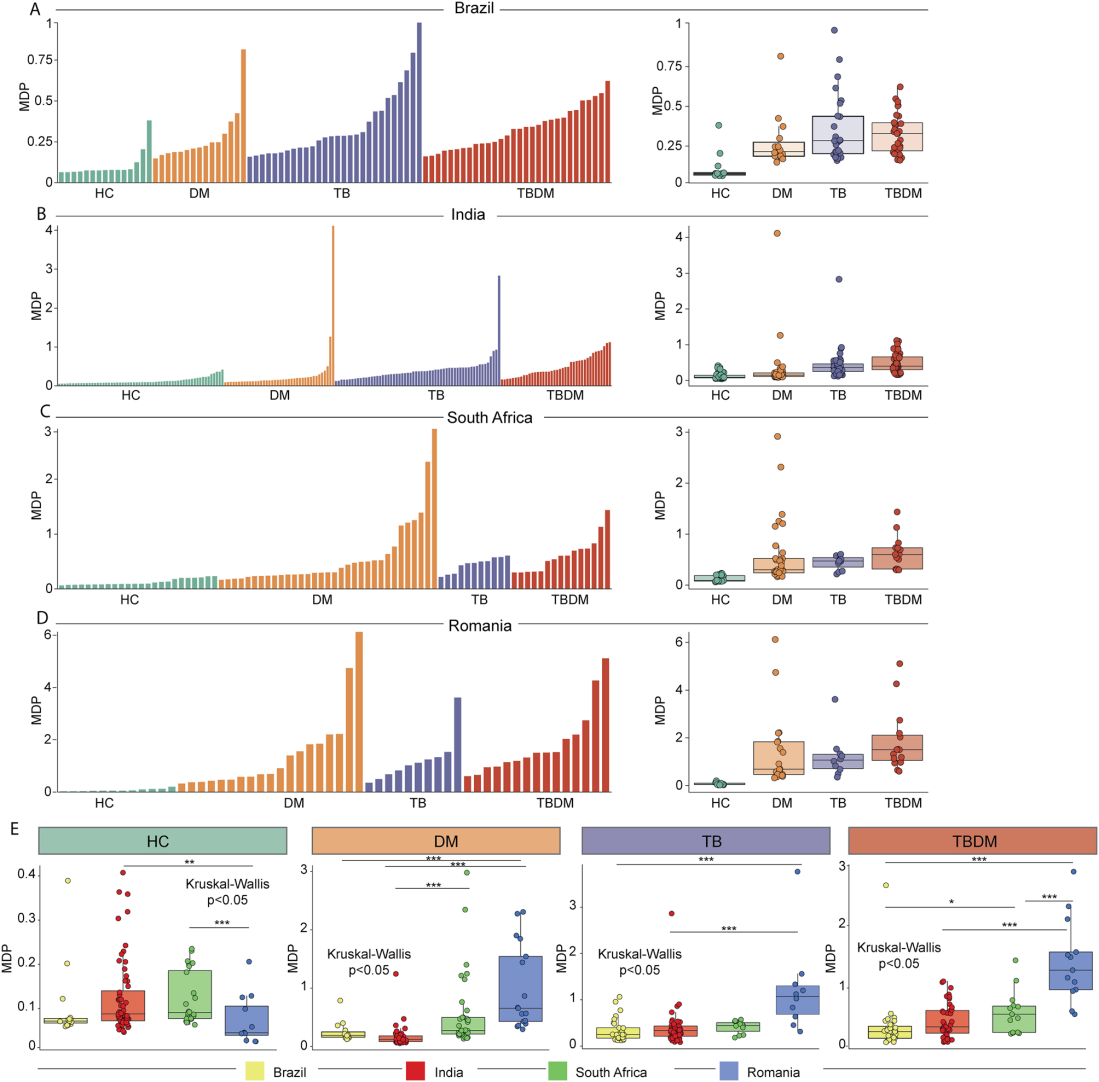
Molecular degree of perturbation stratified by clinical group and site. Histograms show molecular degree of perturbation (MDP) scores of each sample (left) and boxplots of each group (right) are shown for Brazil (**A**), India (**B**), South Africa (**C**) and Romania (**D**), using Kruskal–Wallis test. (**E**) Boxplots of MDP by of each clinical group (DM, TB, TBDM and HC) compared between sites. To estimate which groups are different from others, Tukey’s post test was employed. Thus, *refers to p ≤ 0.05, **refers to p ≤ 0.01, ***refers to p ≤ 0.001 and ****refers to p ≤ 0.0001.

Gene expression within the disease condition groups (DM, TB, TBDM) was compared against site-specific HC participants. The raw data on DEGs are shown in Supplementary File S1. Across all four sites, the DM groups exhibited no DEGs in common, while the TB and TBDM groups shared six and twelve DEGs, respectively (Fig. 1A). A z-score normalized heatmap using the combined DEG gene expression values further demonstrated the variability within and between groups and sites, finding no pattern that classified any clinical group across all sites (Fig. 1B). A principal component analysis model (PCA) applied to DEGs differentiated between groups by the presence or absence of TB disease but did not discriminate between TB and TBDM (Supplementary Fig. S2A,B). Furthermore, we calculated the molecular degree of perturbation (MDP) score^10^ to estimate the overall level of inflammation within disease groups at each site. The highest individual and median MDP values were present in the TB and TBDM groups and there was a non-significant trend for higher MDP in the TBDM than in the TB groups at all sites (Fig. 2A–D). Statistically significant differences in MDP scores across the four study sites were identified in the HC, DM, TB, and TBDM groups (Fig. 2E). To evaluate possible effects of clinical and epidemiological features in the differential gene expression, we performed a PCA, labelling the participants according to presence or absence of cavitation (Supplementary Fig. S3A,B). Furthermore, a Spearman correlation between BMI and the MDP values was performed (Supplementary Fig. S3C,D). No association between BMI values and gene expression variation was observed in the TB and TBDM groups in both sites. The results showed that we could not segregate the participants according to the presence or absence of cavitation, and that the BMI values were not associated with the degree of inflammatory activation, highlighting that the population-specific differences for all these clinical conditions were likely not associated with clinical and epidemiological features.

### Discovery and validation of a TB-associated gene expression signature

**Figure 3:**
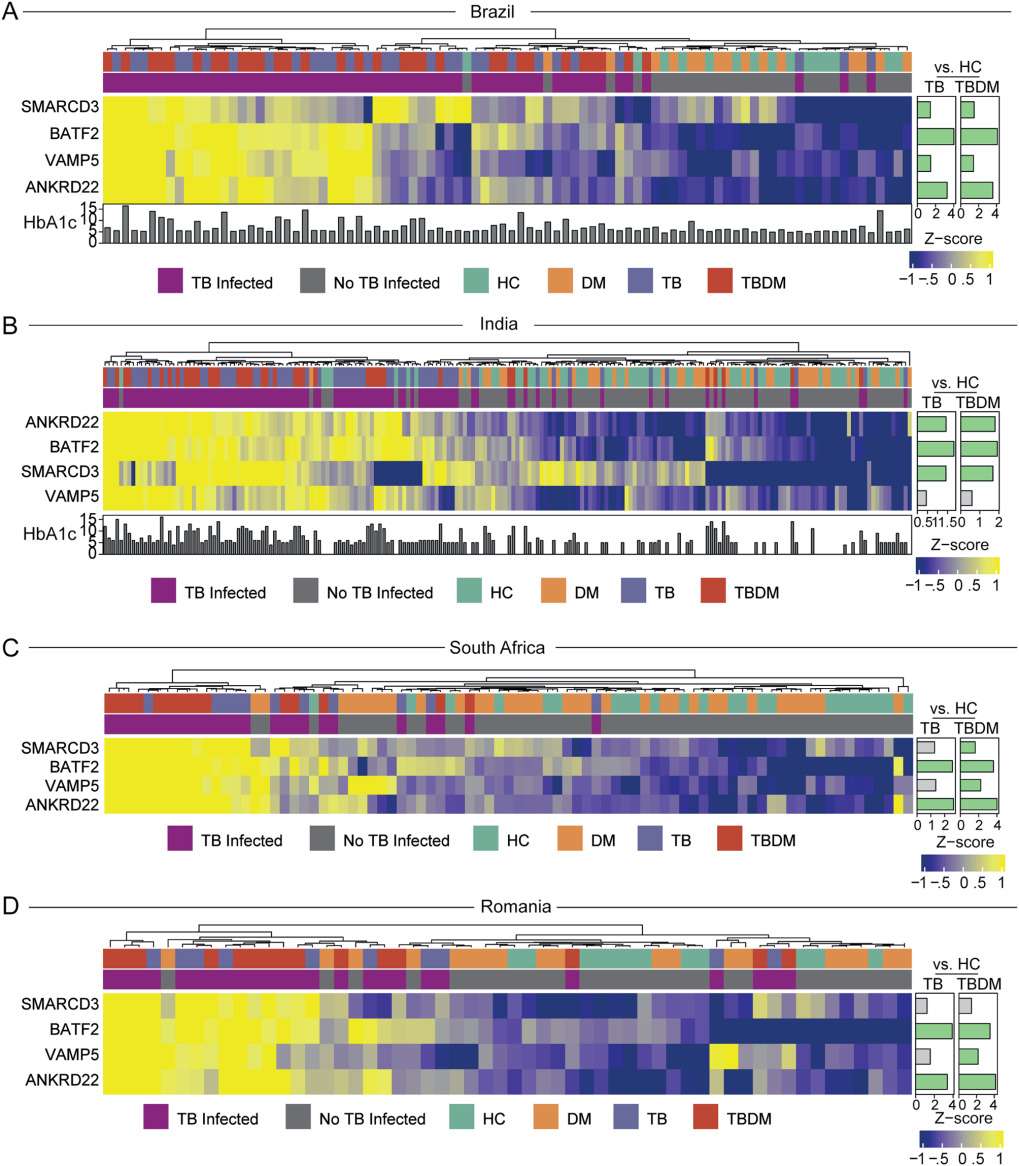
Relative expression of four TB signature genes at each site. Using a machine learning analysis, we defined four top genes among the clinical sites (as showed in Supplementary Material). A z-score normalized heatmap was employed to depict overall trends in gene expression among the clinical groups each study site, as indicated. Panels to the right of heatmaps show the average fold-difference between the signature gene expression in the HC group versus TB and TBDM (log-transformed values).

**Figure 4:**
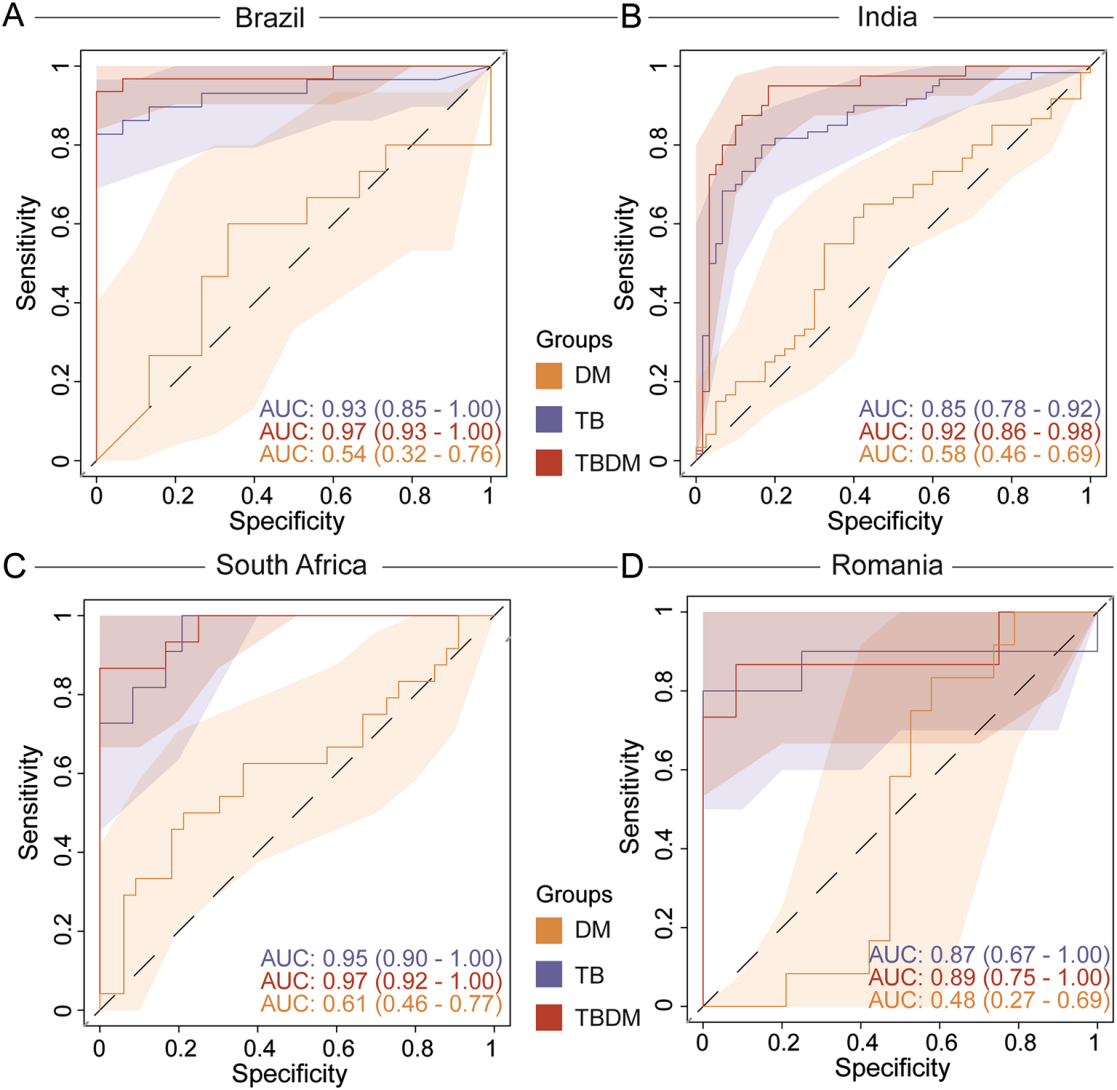
Accuracy of top the 4-gene signature to classify TB, with or without DM. Receiver operator curve (ROC) analysis was used to check the accuracy of the signature genes identified by the random forest model to classify the TB, TBDM, and DM groups in each clinical site as indicated with respect to TB disease.

**Figure 5:**
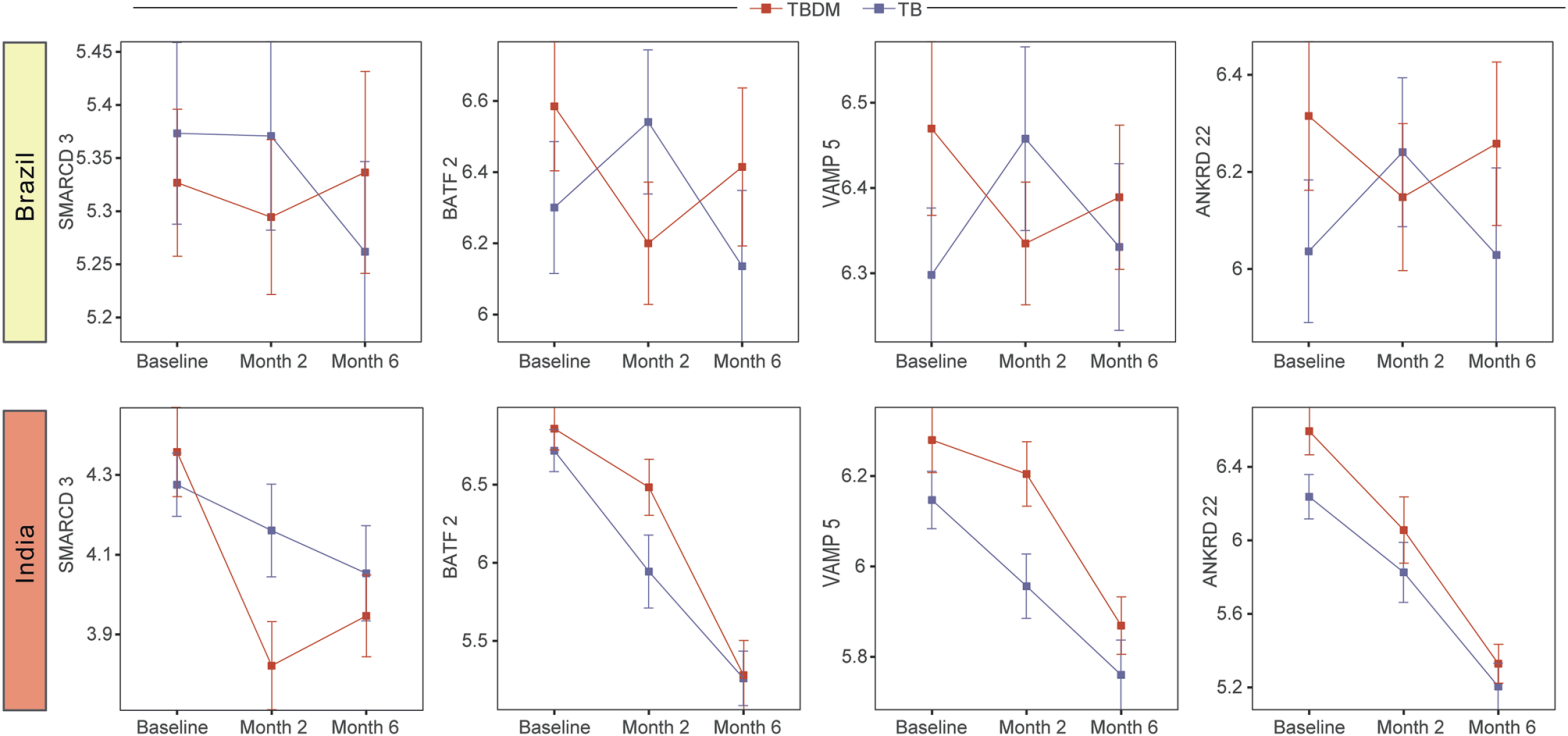
A distinctive pattern of gene expression during TB treatment. Expression of the four TB signature genes was analyzed at baseline, month-2 and month-6 in MSTDI participants in India and Brazil. Nemenyi’s non-parametric all-pairs comparison was used.

As an alternative approach to identify gene expression patterns associated with specific groups across all sites, we inputted expression values of the combined total of 3427 DEGs for a random forest model (Supplementary Fig. S4). The South Africa and Romania sites were used as a discovery set for each of the four conditions (HC, DM, TB, TBDM) and the model was tested in a validation set composed of samples from India and Brazil (Supplementary Fig. S4). Model validation resulted in the accuracy of 58.97 (63.32–74.84); *p* = 0.001658). Gene expression data were used in a feature selection analysis with a random forest algorithm to rank the variables according to their model importance and those above the third quartile were selected, identifying SMARCD3, VAMP5, ANKRD22, and BATF2 as most informative genes to distinguish the clinical conditions (Supplementary Fig. S4A). An unsupervised cluster analysis of z-score normalized expression data identified higher baseline expression of these four genes in participants with TB at all four sites, irrespective of DM status (Fig. 3). Consistent with that observation, Spearman correlation analysis performed between HbA1c levels and expression of the most informative genes did not reveal a consistent association between the expression of these genes and HbA1c levels in participants from Brazil or India (Supplementary Fig. S4B,C). While the 4-gene signature did not discriminate between TB and TBDM, receiver operator characteristic (ROC) curve analysis demonstrated that the signature presented good accuracy to classify TB and TBDM from DM and HC at all four sites with ROC ≥ 0.85 (Fig. 4). Additionally, we tested the accuracy of previously published gene biosignatures in our study sites, with relatively high accuracy of most of signatures to identify either TB or TBDM participants (Supplementary Figs. S5, S6, respectively). The temporal expression of these signature genes over the course of TB treatment differed among the individual genes, the condition (TB or TBDM), and between the Indian and Brazilian cohorts (Fig. 5). Expression levels of all four signature genes tended to be higher in TBDM than TB in the Brazil cohort at month-6, rising from a nadir at month-3. In the India cohort, expression of BATF2, VAMP5, and ANKRD22 tended to be higher in TBDM than TB at baseline and month-2, with VAMP5 and ANKRD22 continuing that trend to month-6. These patterns might reflect persistent inflammation in TBDM, which was identified in prior study measuring plasma cytokines^11^.

### Pathway enrichment and interaction analysis across conditions and populations

**Figure 6:**
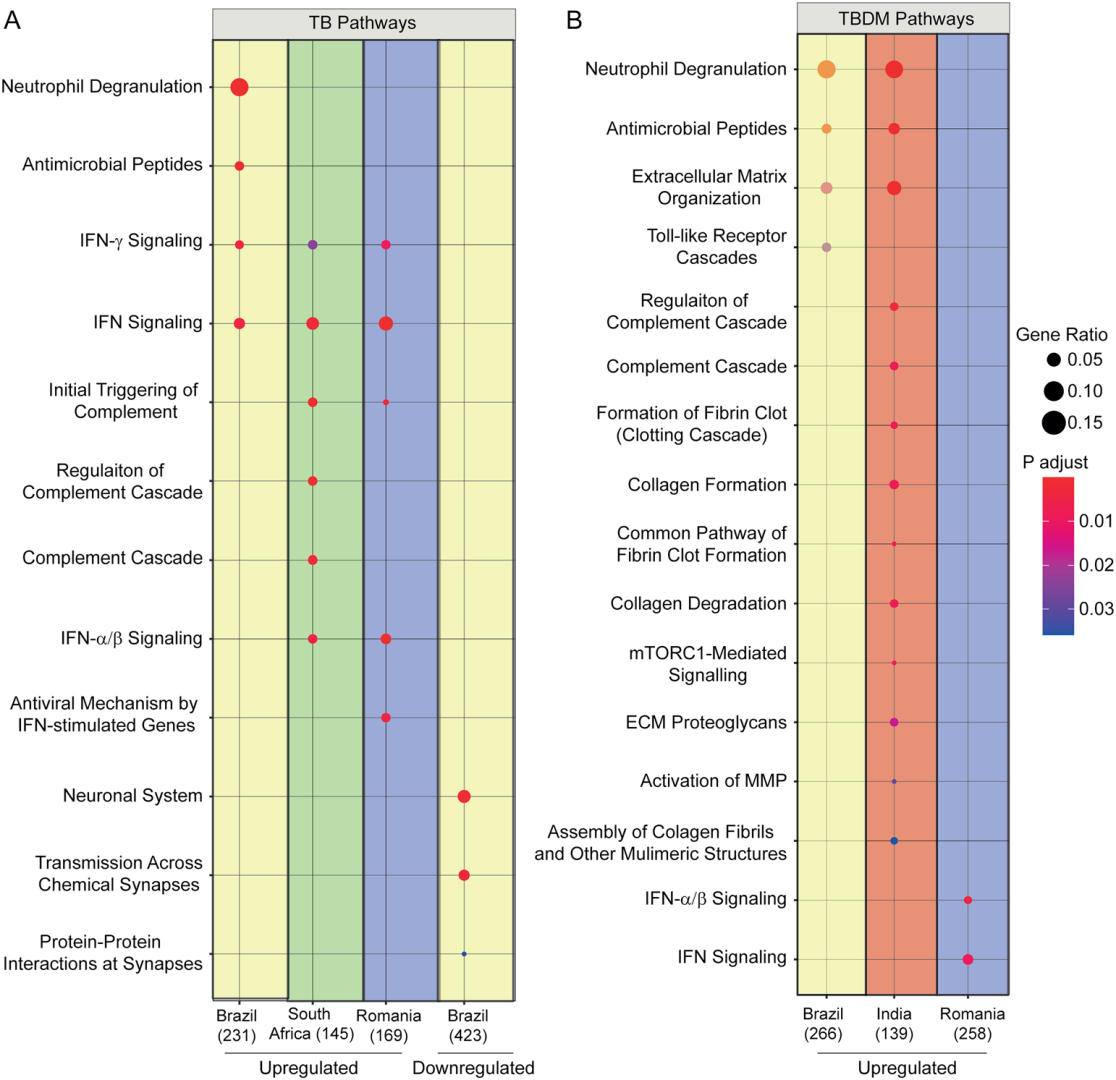
Pathway enrichment by condition and site. The colored spots indicate selected upregulated and downregulated Reactome pathways of interest identified by the combined significant (p < 0.05) and FDR-corrected DEGs from the TB groups (**A**) and TBDM groups (**B**) in Brazil (yellow), India (red), South Africa (green), and Romania (blue). The hue of spots corresponds to p-value and the area corresponds to gene ratio. The method did not detect any pathways in TB participants from India.

While comparison of DEGs failed to discriminate between TB and TBDM, we questioned whether pathway enrichment had the potential to reveal condition-specific differences. Reactome pathways of interest were identified within significant (*p* < 0.05) and false discovery rate (FDR)-corrected DEGs from all conditions and sites. No pathway was uniquely enriched within the TB or TBDM groups at all sites, but interferon signaling that has been identified in many TB gene expression studies^12^ was enriched among TB participants from Brazil, South Africa, and Romania (Fig. 6A). The India and Brazil TBDM groups shared enrichment of the Neutrophil.

Degranulation, Antimicrobial Peptides, and Extracellular Matrix Organization pathways. Interferon signaling was enriched only in the Romanian TBDM group but not in TBDM participants from the other sites (Fig. 6B). Mirroring the DEG analysis, the changes in pathway predominance during TB treatment were markedly different between sites (Supplementary Fig. S7). Although pathway enrichment did not consistently distinguish TBDM from TB across all sites, the trend for increased Neutrophil Degranulation, Antimicrobial Peptides, and Matrix Organization fits with prior reports of neutrophilic inflammation and elevated circulating levels of cathelicidin, human beta defensin- 2, human neutrophil peptides 1–3, and matrix metalloproteinases in TB-DM comorbidity^7,13,14^.

**Figure 7:**
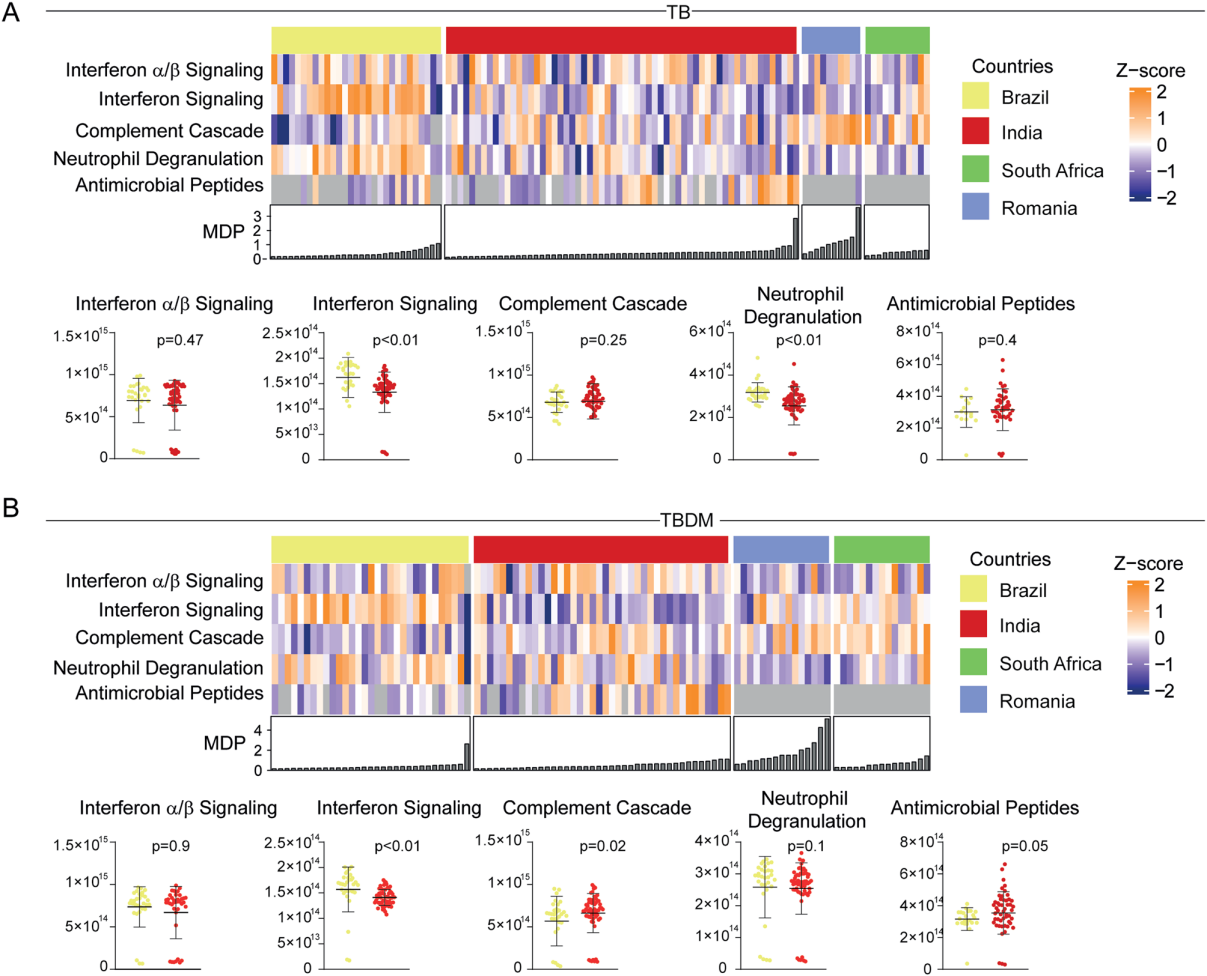
Changes in the pathways expression across clinical sites and diseases. A heatmap Z-score normalized was employed using the overlapped pathways identified in the enrichment analysis from DEGs (as described in Fig. 3). Box plots represents median and interquartile ranges, Mann Whitney test was used.

The Neutrophil Degranulation, Antimicrobial Peptides, Interferon Signaling, Regulation of Complement Cascade, Complement Cascade, and Interferon alpha/beta Signaling pathways were evaluated using a hierarchical cluster analysis ordinating participants by MDP levels in TB and TBDM groups (Fig. 7A,B). This analysis was limited by the absence of some pathway enrichment data from South Africa and Romania, thus box plots from only Brazil and India are displayed. We observed a higher expression of the Interferon Signaling and Neutrophil Degranulation pathways in Brazilian TB participants when compared with the TB group from India (Fig. 7A). Among TBDM groups, the Interferon Signaling pathway was more highly expressed in Brazil while the Complement Cascade pathway was more highly expressed in India (Fig. 7B).

**Figure 8:**
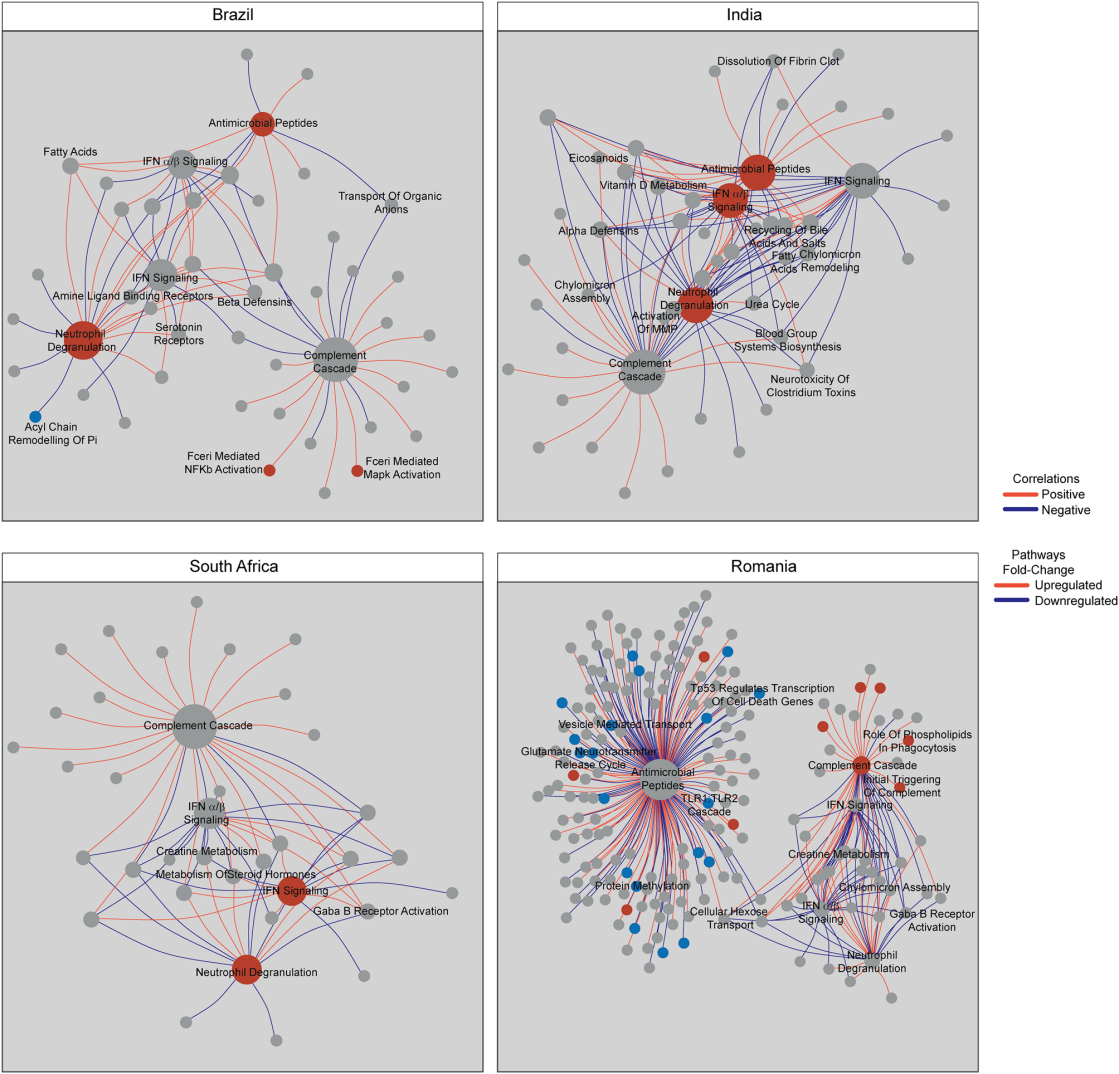
Pathway dynamicity across the sites and disease groups. A Spearman correlation analysis was performed using the pathways from TBDM participants in each clinical site, as indicated. Each node indicates a pathway, blue nodes indicate downregulation compared to the site-specific HC group, while red nodes indicate upregulation. Grey nodes represent pathways that were not statistically significant compared to the site-specific healthy control group. Red lines infer positive correlation and blue lines negative interaction.

To further evaluate the interaction between DM and TB, we examined the interactions between pathways identified from DEGs compared to Reactome in the TBDM groups (Fig. 8) and TB groups (Supplementary Fig. S8) which were observed at least two sites. This approach differed from our initial pathway analysis by estimating enrichment scores computed in each sample instead of a set of DEGs, enabling further analyses such as correlation or fold change score. Correlation network analysis and fold-change analysis using single sample gene set enrichment analysis (ssGSEA) to generate normalized enrichment scores (NES) identified differences across sites and clinical groups, along with highly correlated pathways at some sites that were not identified by the prior enrichment analysis (Fig. 6), which was not suitable to investigate correlation. As shown in Fig. 8, higher density of network pathways in TBDM was found in South Africa (0.108), India (0.091), and Brazil (0.069). Network pathway density was markedly lower in the Romanian TBDM group (0.013) despite a higher number of vertices found in these correlations (Fig. 8). Excluding the Romanian cohort, the top correlated pathway in TBDM was Complement Cascade, with 22 connections in Brazil, 30 correlations in India and 25 in South Africa, however, the pathway was not uniformly up or downregulated in TBDM at all the three sites compared with the site-specific healthy control group. Correlation analysis for the normoglycemic TB groups using ssGSEA-NES values showed comparable diversity between sites (Supplementary Fig. S8).

### Correlation of ssGSEA pathways with HbA1c

**Figure 9:**
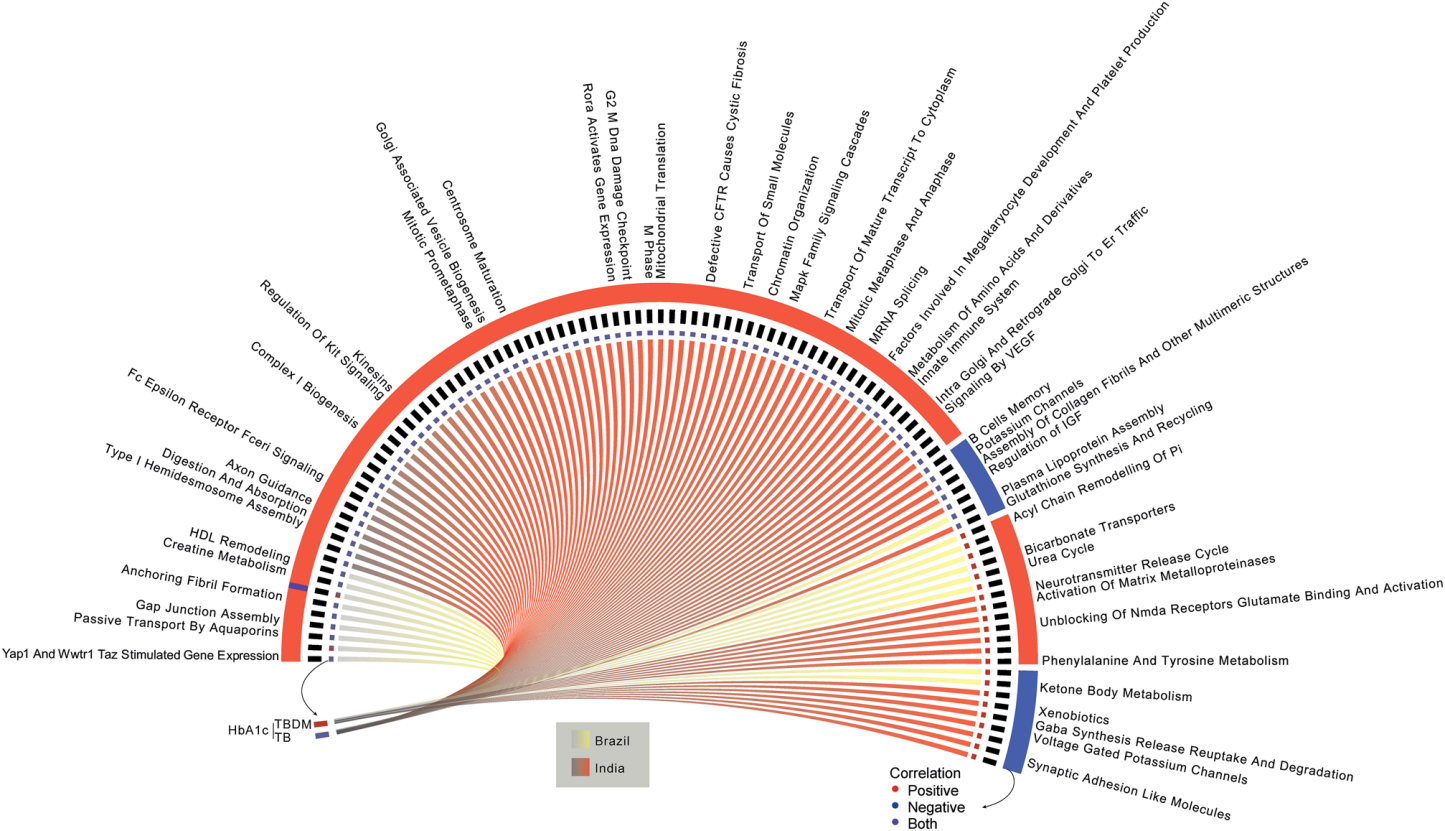
Integrative analysis of HbA1c and Pathways expression in Brazil and India. Levels of HbA1c were correlated with the pathways according to disease group as indicated in the left box (red refers to TBDM whereas purple to TB). The external-colored bars indicated the direction of correlation, as indicated in right circles (red for positive correlation, blue for negative correlation, and purple for positive and negative correlations). Lines in center of the figure refers to the country (red for India and yellow for Brazil).

Finally, to assess the influence of average blood glucose levels with pathway engagement, we employed a correlation model using HbA1c levels and ssGSEA NES values with available data from Brazil and India (Fig. 9). Among TB group participants, the India cohort data showed a substantially higher number correlations (68 positive and 6 negative) than Brazil (9 positive and only one negative) (Fig. 9). The TBDM comparison showed a lower total number of correlations than was seen for TB, again with more in the Indian cohort (7 positive and 7 negative correlations) compared to Brazil (6 positive and 2 negative correlations) (Fig. 9). There was no overlap in these correlated pathways between the sites or conditions. Notably, many of the positive correlations from the India TB group were for pathways associated with insulin resistance and metabolic syndrome (e.g. Passive Transport by Aquaporins, Yap1 and WWTR1 Taz Stimulated Gene Expression, Complex I Biogenesis, Mitochondrial Translation), diabetic complications (Axon Guidance, Regulation of Kit Signaling, Kinesins, MAPK Family Signaling Cascades, Mitochondrial Translation, VEGF Signaling, Factors Involved in Megakaryocyte Development and Platelet Production, Unblocking of NMDA Receptors Glutamate Binding and Activation), and pathways associated with chromosomal instability (Mitotic Prometaphase, Centrosome Maturation, G2 M DNA Damage, M Phase, Chromatin Organization, Mitotic Metaphase and Anaphase). Correlation with immune and inflammatory pathways were less prominent but included positive correlations with the RORA Activates Gene Expression and the Innate Immune System pathways in the India TB group, and negative correlations with B Cells Memory and Potassium Channels Pathways. The universally high level of HbA1c prevented correlation of these pathways in TBDM individuals.

## Discussion

Whole blood RNAseq potentially offers a window into biological processes at the primary site of TB disease in the lung. In an earlier blood gene expression study at Chennai with a different group of participants, we found no differences in immune response pathway activation between TB and TBDM^7^. To address shortcomings of that study done at one site and using microarrays, the MSTDI study included participants from Chennai and Pune in India and Salvador in Brazil and used RNAseq performed on the same platform. Results of the MSTDI study supported our prior findings. There was no evident signature pattern of immune response gene expression or pathway activation that offered mechanistic insight to the basis of TB susceptibility in people living with DM. However, trends for increased neutrophil and innate immune pathway activation in TBDM were noted. Studies in hyperglycemic mice identified a defect in alveolar macrophage sentinel function as the key immune mechanism of TB susceptibility in DM^15–17^. This impacts the initial encounter of alveolar macrophages with inhaled *M. tuberculosis*, delaying the transition from innate to adaptive immunity during the period of logarithmic increase in lung bacterial load. Once the adaptive immune response is expressed in diabetic mice, it effectively limits *M. tuberculosis* replication and is qualitatively indistinguishable from the response of normoglycemic control mice with TB. If a similar mechanism operates in human DM, then the key immunological events contributing to susceptibility will have occurred months before TB diagnosis. Gene expression at baseline in human studies corresponds to the later time points of mouse experiments when the cell-mediated immune response is fully activated.

Based on animal model data, we anticipated that immune pathology in human TBDM would be qualitatively similar to euglycemic TB but quantitatively more severe^18^. That prediction was supported by plasma cytokine and radiographic studies^7,19^, but not so clearly by differences in whole blood gene expression^7^. The TANDEM study included four geographic sites and reported an overall increase of innate immune gene expression with decreased expression of genes linked to adaptive immunity in participants with TB combined with DM or pre-DM^20^. That result supports the general notion of increased immune pathology in TBDM but does not explain the broadly elevated plasma levels of Th1, Th2, and Th17 cytokines reported in this condition^7,21^. The MSTDI data presented here showed a non-significant trend for higher MDP values in TBDM than TB participants, whereas the differences in MDP between sites were statistically significant. Pathway enrichment, interaction, and correlation analyses likewise failed to identify a universally consistent TBDM-specific pattern of engagement. Overall, the data suggest greater severity of immune pathology in TBDM, which more likely reflects a consequence rather than cause of TB susceptibility in people living with DM.

Results from the MSTDI study, notably the correlation of pathway engagement with HbA1c levels, further support the potential for TB to exacerbate non-communicable disease processes^7,9^. Bacterial pneumonia and COVID-19 are both associated with increased risk of cardiovascular disease events^22,23^. A similar association has been made with TB^24^ where the longer duration of inflammation, particularly with comorbid DM^11^, could be particularly damaging. Similar reasoning would apply to the microvascular complications of DM, but this has not been studied.

An unexpected finding in our analyses was the high degree of variability in baseline gene expression between all four populations evaluated. Of note, some differences in the clinical presentation and disease severity were found in our study, as in TB, with a higher percentage of cavitations in India when compared with Brazilian TBDM participants, as well in DM, with higher HbA1c levels detected in India. The heterogeneity in this proportion could affect the gene expression and to verify its influence we performed an additional analysis that demonstrated an apparent lack of association between such parameters and differences in gene expression profiles among the countries. Equally remarkable were the different temporal patterns of gene expression in the India and Brazil cohorts from baseline through TB treatment completion at month 6. This variability presumably reflects differences in host and microbe genetics, behaviors, and environmental exposures between sites. Similar issues have hampered the application of concise gene expression signatures for TB diagnosis and treatment response at different sites^25^. Further research will be required to identify the fundamental processes influencing the TB-DM interaction and their expression in diverse populations.

This work has some limitations. First, not all clinical and epidemiological data are available from all datasets used. The data from South Africa and Romania did not present other information such as HbA1c levels, Cavitation or alcohol, and smoking usage. For this reason, only age and sex were used to adjust the models in differential expression analysis. The second limitation was the differences observed in the BMI values, age, sex, smoking, and alcohol use between the population of Brazil and India (Supplementary Tables S1 and S2). Additionally, some patients were under treatment with metformin and statin, that could affect the inflammatory responses. We have applied a negative binomial model for the differential gene expression analysis. This modeling has limitations regarding multiple variable adjustments. Despite we have evaluated the Cavitary TB and BMI association with gene expression, their systemic influence could not be fully corrected in the model. Moreover, use of metformin and/or statins, and frequency of cavitary TB were significantly different in TB-infected individuals between those countries. Cavitary TB and BMI values are known to affect immune activation and thus could influence the gene expression profile of those subjects and may explain the absence of overlapping pathways in Brazil and India sites. Another limitation was associated with the different sample sizes observed in the cohorts (Supplementary Fig. S1). South Africa data presented 11 TB and 15 TBDM samples, Romania 10 TB and 15 TBDM samples, Brazil 29 TB and 31 TBDM samples, and India 60 TB and 40 TBDM samples. This unbalanced sample size could have influenced both gene expression analysis and signature performance. DM is a metabolic disease, and it is reasonable to speculate that using other platforms, such as metabolomics, could increase the odds of detecting discrepancies between TB and TBDM. We are currently performing new investigations using multi-omics to portray the TB-DM interaction.

This work has some differences compared with the TANDEM work which makes the straightforward comparison of the results difficult. First, the TANDEM has used data from 4 different sites (South Africa, Romania, Peru, and Indonesia). However, all the analysis was performed with whole data, without performing the comparisons within the sites, as was performed in the MSTDI. Also, two TANDEM sites do not have healthy controls, thus all comparisons from the case subjects from Peru and Indonesia were performed with foreign control, which may have inserted variation. Second, the enrichment analysis was performed using different approaches. In TANDEM, the transcriptional module enrichment analysis was performed with tmod package while two different approaches were performed in the MSTDI, using clusterProfiles and Reactome database, and the single sample gene set enrichment analysis with ssGSEA package. This shows that the cohorts explored the disease dynamics in a different optic, with the first exploring the overall impact and the second one investigating the populational contribution on this impact.

In summary, we found substantial variability in whole blood gene expression between HC, DM, TB and TBDM participants across the two MSTDI study sites in Brazil and India and the TANDEM study South African and Romanian cohorts. No mechanistically informative signature of immune pathway gene expression distinguished TBDM vs TB in all populations, although increased innate immune and vascular complication pathway activation was common across some sites. Our findings lend evidence in support of adjunctive anti-inflammatory and antioxidant therapies during TB treatment. In that regard, retrospective evidence demonstrated that metformin added to antibiotic treatment reduces the mortality risk in TBDM independent of glycemic control^26^ and a recently completed randomized controlled trial of TBDM patients in India showed that metformin reduced inflammation and radiographic severity in disease^27^.

## Materials and methods

### Ethics statement and study population

The MSTDI study used unpublished data and samples from the RePORT India and the RePORT Brazil consortia. Samples from the RePORT India were enrolled under protocols approved by the Ethics Committee of the Prof. M. Viswanathan Diabetes Research Center and the Institutional Review Boards of Byramjee Jeejeebhoy Government Medical College, Pune and National Institute for Research in Tuberculosis and Johns Hopkins University. The samples enrolled from the RePORT Brazil had their protocols approved by the institutional review boards of the Instituto Gonçalo Moniz, Fundação Oswaldo Cruz and Vanderbilt University Medical Center. Written informed consent was obtained from all participants prospectively enrolled at two sites of the RePORT India and one site of the RePORT Brazil consortia, with organizational support from RePORT International^28^. The study was conducted according to the principles of the Declaration of Helsinki. The Indian sites were in Chennai (EDOTS study)^29^ and Pune (CTRIUMPH study^30^), while the Brazilian site was in Salvador (RePORT International Common Protocol)^28^. The combined MSTDI cohort of 290 individuals comprised 120 participants from Chennai, 80 from Pune and 90 from Salvador. These participants were selected from a larger cohort of the RePORT protocols based of sample availability. Participant groups included pulmonary TB disease with or without DM (TBDM and TB groups, respectively) and two control groups without TB, with or without DM (DM and HC groups, respectively). Inclusion criteria were age 18–65 and new diagnosis of pulmonary TB (or absence of pulmonary TB for the control group participants). Drug-resistant TB, retreatment TB, treatment of incident TB for > 7 days prior to enrollment, pregnancy, immunosuppressive medications, and HIV infection were exclusions. All TB data used here were obtained from participants accompanied during the 6 months of anti-TB therapy according to the RePORT common protocol and who were successfully treated according to WHO criteria^31^. Participant characteristics are presented in Supplementary Tables S1 and S2 of the Supplementary Material. Secondary data were used from the TANDEM study^32^ that explored the TB-DM interactions at clinical sites in Indonesia, Peru, Romania, and South Africa. Patients from TANDEM were also on TB treatment according to the respective local TB program. We used TANDEM data from South Africa and Romania where site-specific healthy control participant data were available. This data set comprised 83 participants from South Africa (24 HC, 33 DM, 11 TB, 15 TBDM) 56 from Romania (12 HC, 19 DM, 10 TB, 15 TBDM). The group sizes for all sites used in our analysis are summarized in Supplementary Fig. S1.

### Classification and blood sampling

TB diagnosis was based on positive sputum culture for *M. tuberculosis* with a compatible chest x-ray at enrollment, while negative culture and x-ray defined the control groups. Classification with DM was based on self-reported medical and anti-diabetic medication history or glycohemoglobin (HbA1c) ≥ 6.5%. Classification as euglycemic was based on self-reported medical history and HbA1c < 5.7% or 75-g oral glucose tolerance test 2-h blood glucose < 140 mg/dL. Baseline blood samples were collected in RNA storage tubes from all participants at the time of enrollment and no later than 7 days after the initiation of anti-tubercular treatment.

### Library preparation and RNA sequencing

The samples from TB-infected individuals from both India and Brazil sites were collected at baseline, 2, and 6 months of treatment. Samples for the HC and DM groups were collected at baseline. Whole blood (5 mL) was collected PAXgene Blood RNA tubes (Qiagen, catalog #762165) and frozen at − 80 °C. RNA was extracted using the PAXgene Blood RNA kit (Qiagen, catalog #762174) and quantified using Qubit RNA assay HS (Invitrogen, Cat #Q32852). RNA purity was checked using QIAxpert, and RNA integrity was assessed on TapeStation using RNA HS ScreenTapes (Agilent, Cat #5067-5579). NEB Ultra II Directional RNA-Seq Library Prep kit protocol was used to prepare libraries for total RNA sequencing. Prepared libraries were quantified using Qubit High Sensitivity Assay (Invitrogen, Cat #Q32852), pooled and diluted to final optimal loading concentration before cluster amplification on Illumina flow cell. Once the cluster generation was completed, the cluster flow cell was loaded on Illumina HiSeq 2500 instrument to generate paired end reads at MedGenome in Bangalore, India.

### RNA-seq data analysis

Raw RNA-seq data from the MSTDI cohort were retrieved from Illumina HiSeq 2500 platform. Sequence data from the TANDEM cohort^8^ was retrieved from the SRA database using BioProject PRJNA470512 using the SRA tools. Sequence data from MSTDI and TANDEM were quality control processed by removing low-quality bases and adapters using *Trimmomatic V0.32*. After the quality check, sequences were pseudo-aligned against the human transcriptome (GRCh38 version) comprising both mRNA and miRNA with *salmon* v0.8.2^33^ and presented a mean mapping rate of 68.03 ± 2.28. After mapping, the output was converted to a count table using *tximport* package^34^ from *R 4.1.3*. Count gene expression matrix was examined using the *edgeR* package^35^ from *R4.1.3* to identify differentially expressed genes (DEGs). MSTDI gene expression data are available at the GEO database (Accession number GSE181143, https://www.ncbi.nlm.nih.gov/geo/query/acc.cgi?acc=GSE181143).

Differential expression analysis in the MSTDI cohort compared three conditions (TB, TBDM, DM) at two sites (Brazil and India) to the healthy control (HC) group at the respective site to determine the fold change and *p*-value of each gene. For instance, the TB, TBDM, and DM subjects from one country are compared with the HC subjects from the same country, and not from another country, to avoid inserting operational variation in the analysis. From the TANDEM cohort, only two sites (South Africa and Romania) were used in the differential expression analysis. The other sites (Peru and Indonesia) lack the HC group at the respective sites. Multiple testing correction was performed using the Benjamini & Hochberg false discovery rate (FDR) method^36^. Changes in gene expression were considered significant when corrected *p*-values remained < 0.05 after FDR adjustment and if the fold change differences were higher than ± 1.4. The complete list of genes is available in Supplementary File 1. The DEGs were visualized using the *VennDiagram* package^37^ from *R.4.1.3*. The *compareCluster* package^38^ from *R4.1.3* was used with the obtained DEGs to scan the *REACTOME* pathway database^39^ to perform the pathway enrichment analyses and the the Benjamini & Hochberg false discovery rate (FDR) method^36^ was used to correct the p-values for multiple testing.

Participant characteristics tables present the median and interquartile range or percentage for the nominal variables. All comparisons were performed with Kruskal–Wallis with Tukey’s post test and Chi-squares tests using R*.4.1.3*.

### Population heterogeneity evaluation and feature selection analysis

Sample variation within and between sites was evaluated using the molecular degree of perturbation (MDP) package^10^ applied in the gene expression values after variance-stabilizing transformation (VST), for Brazil, South Africa and Romania data, and batch effect correction with sva package^40^ in the case of India site data. A gene was classified as perturbed when its variation compared to HC was > 2 standard deviations.

To evaluate the sample clustering and classification across the sites we performed one-sided unsupervised hierarchical clustering Ward’s method^41^, Heatmaps^42^ and the Principal component analysis (PCA) plot in the VST gene expression values from each cohort (Supplementary Figs. S2, S3A,B). This approach allows the visualization of sample dispersion across the groups in the sites. To maximize the performance in the TB and TBDM classification, we employed a dimensionality reduction approach to reduce the number of genes associated with TB. Thus, we retrieved the VST gene expression of all 3427 DEGs from the comparisons. The data from South Africa and Romania were used as a discovery set in a random forest algorithm with leave-one-out cross-validation with the *caret*^43^ and *randomForest*^44^ packages. The data from Brazil and India was used as a validation set (Supplementary Fig. S4). The minimal gene set exhibiting higher classification power to describe the groups was defined by the variable importance in the random forest model. Gene expression values from each were retrieved from each site and the Receiver Operator Characteristics (ROC)^45^ were used to assess the accuracy of the gene set to distinguish between comparison groups specified in the TANDEM and MSTDI datasets.

### Performance analysis with previously identified signatures

We conducted a performance comparison using 69 previously published gene expression signatures for TB diagnosis, progression, and treatment provided by the *TBSignatureProfiler* package (https://github.com/compbiomed/TBSignatureProfiler). In addition, we have included RISK6, RISK11 and BATF2 signatures for comparison (Supplementary Figs. S5 and S6). The signature classification performance was performed using data from each signature data from TB and HC (Supplementary Fig. S5), and TBDM and HC (Supplementary Fig. S5) within each country, similar to the analysis performed to identify the DEGs. We estimated the area under the curve (AUC) values of each signature with its confidence interval (CI), by applying a general linear model to gene expression values from each comparison (TB and HC or TBDM vs HC, within the countries). The detailed performance of each signature in each clinical conditions is shown in the Supplementary File S2. Moreover, linear modeling allows the comparison of performance from signatures composed by DEGs and composed by scores, as well as a fair comparison between them^46,47^. The outcomes were binarized to measure the sensitivity and specificity of classification, allowing us to measure each group rate and plot each signature AUC and CI value, from each country. This allows the direct performance comparison of each signature in data from different countries and different conditions (TB or TBDM).

### Single sample gene set enrichment analysis (ssGSEA)

The normalized enrichment scores (NES) from each sample were calculated with ssGSEA^48^ using the Reactome database^39^. Only significant NES values were used (FDR < 0.05 and 100 permutations) to perform correlation analysis with the enriched pathways, HbA1c values and gene expression levels. Correlation relationships were depicted as chord diagrams and networks, performed by *circlize*^49^ and *igraph*^50^ package.

## Supplementary Information

Supplementary Information 1.

Supplementary Information 2.

Supplementary Information 3.

## Data Availability

The dataset from the TANDEM cohort analyzed during the current study is available at the BioProject data repository, identified by the accession code PRJNA470512 (https://www.ncbi.nlm.nih.gov/bioproject/?term=PRJNA470512). The MSTDI gene expression data is available at the geoNCBI data repository, identified by the accession number GSE181143 (https://www.ncbi.nlm.nih.gov/geo/query/acc.cgi?acc=GSE181143).
