# Supplementary material for: A multi-center, prospective cohort study of whole blood gene expression in the tuberculosis-diabetes interaction
Source: Sci Rep. 2023 May 12;13:7769. doi: 10.1038/s41598-023-34847-9 (PMC10180618; doi:10.1038/s41598-023-34847-9)
Supplement: Supplementary file 3 — Supplementary Information 3. [file 41598_2023_34847_MOESM3_ESM.docx]

Supplementary Material

| Country | Healthy controls | DM | TB | TBDM | P-value |
| --- | --- | --- | --- | --- | --- |
| Brazil  India | 15 | 15 | 29 | 31 |  |
|  | 60 | 40 | 60 | 40 |  |
| Total (N) | 75 | 55 | 89 | 71 |  |
| Age – y | 35  (30-42) | 54  (44-59) | 36  (28-46.5) | 48  (40-55) | **<0.001** |
| Female – no. (%) | 47  (62%) | 24  (43%) | 25  (28%) | 15  (21%) | **<0.001** |
| BMI (Kg/m²) | 20.9  (15.3-24.5) | 27.9  (24.8-30) | 18.7  (17.3-21) | 22.2  (19-27.3) | **<0.001** |
| Smoking (current) | 7  (9%) | 13  (23%) | 23  (25%) | 18  (25%) | **0.03** |
| Alcohol (current) | 24  (32%) | 23  (42%) | 42  (47%) | 33  (46%) | 0.19 |
| Metformin | N/A | 26  (47%) | N/A | 33  (46%) | 0.9 |
| Statin | N/A | 3  (5%) | N/A | 9  (12%) | 0.22 |
| Cavitary TB | N/A | N/A | 37  (41.5%) | 25  (35.2%) | 0.42 |
| HbA1c (%) | 5.1  (5-5.5) | 9  (7.7-11) | 5.4  (5-5.7) | 10.9  (8-12.1) | **<0.001** |

**Supplementary Table S1:** Characteristics of study population according to clinical groups among the sites of study. Data represent medians and interquartile ranges (age, BMI and HbA1c) and frequencies (female gender, smoking current use, alcohol current use, metformin and statin use and cavitary TB). The Kruskal-Wallis test was used to compare distributions of age, BMI and HbA1c while the Chi-square test was used to compare frequencies of gender, smoking current use, alcohol current use and cavitary, whereas Fisher Exact test used to compare frequencies of metformin and statin use TB. P-values in bold font are statistically significant. Abbreviations: BMI, Body Mass Index; HbA1c, glycosylated hemoglobin, N/A, Non-Applicable.

|  | Healthy controls | |  | DM | |  | TB | |  | TBDM | |  |
| --- | --- | --- | --- | --- | --- | --- | --- | --- | --- | --- | --- | --- |
|  | Brazil | India |  | Brazil | India |  | Brazil | India |  | Brazil | India |  |
| N | 15 | 60 | P-value | 15 | 40 | P-value | 29 | 60 | P-value | 31 | 40 | P-value |
| Age – y | 35  (28-51) | 35  (31-39.7) | 0.64 | 56  (51-57) | 52.5 (42.5-78) | 0.46 | 29  (25-43.5) | 39.5  (31-67) | **0.002** | 48 (38-67) | 48 (40.2-65) | 0.7 |
| Female – no. (%) | 9  (60%) | 32  (53%) | 0.6 | 8  (57%) | 22  (55%) | 0.88 | 10  (34%) | 15  (24%) | 0.35 | 9  (29%) | 10  (25%) | 0.7 |
| BMI (Kg/m²) | 24.9  (20.5-28.9) | 16.9  (16-20) | 0.2 | 30.3  (26-32) | 25.4  (23.6-27.7) | **<0.001** | 20.6  (18.6-22.1) | 16.9 (16-20) | **<0.001** | 22.5  (20-25.7) | 21.9  (18-28.9) | 0.79 |
| Smoking (current) | 5  (33.3%) | 2  (3.3%) | **0.004** | 3  (20%) | 10  (25%) | 0.6 | 8  (27.6%) | 15 (25%) | 0.7 | 12  (38.7%) | 6  (15%) | **0.02** |
| Alcohol (current) | 13  (86.7%) | 11  (18.4%) | **<0.001** | 14  (93.4%) | 9  (22.5%) | **<0.001** | 25  (86.2%) | 17 (28.4%) | **<0.001** | 28  (90.3%) | 5  (12.5%) | **<0.001** |
| Metformin | N/A | N/A | N/A | Not  assessed | 26  (65%) | Not assessed | N/A | N/A | N/A | 6  (19.4%) | 27  (87%) | **<0.001** |
| Statin | N/A | N/A | N/A | Not  assessed | 3  (7.5%) | Not assessed | N/A | N/A | N/A | Not assessed | 9  (22.5%) | **<0.001** |
| Cavitary TB | N/A | N/A | N/A | N/A | N/A | N/A | 15  (51.7%) | 22 (36.7%) | 0.17 | 9  (29%) | 26  (65%) | **<0.001** |
| HbA1c (%) | 5.1  (4.9-5.2) | 5  (5-5.5) | 0.25 | 6.1  (5.9-7.4) | 9.4  (8.4-11.1) | **<0.001** | 5.5  (5.2-5.6) | 5.3  (5-5.9) | 0.84 | 8.5  (6.8-11.4) | 11.7  (10-12.5) | **0.001** |

**Supplementary Table S2:** Characteristics of study population according to clinical groups among the sites of study. Data represent medians

and interquartile ranges (age, BMI and HbA1c) and frequencies (female gender, smoking current use, alcohol current use, metformin and statin

use and cavitary TB). The Kruskal-Wallis test was used to compare distributions of age, BMI and HbA1c while the Chi-square test was used to

compare frequencies. P-values in bold font are statistically significant. Abbreviations: BMI, Body Mass Index; HbA1c, glycosylated hemoglobin,

N/A, Non-Applicable.

**
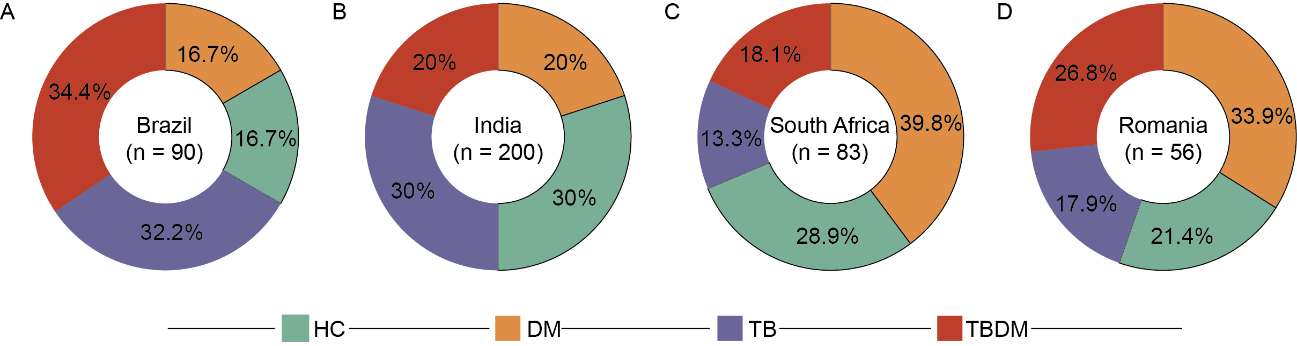
**

**Supplementary Fig. S1.** Sample distribution in the MSTDI and TANDEN cohort. Colored sections show the percentage of individuals at (A) Brazil, (B) India, and (C) South Africa and (D) Romania in the healthy control group (HC), diabetic control group (DM), and the non-diabetic and diabetic pulmonary TB groups (TB and TBDM, respectively).


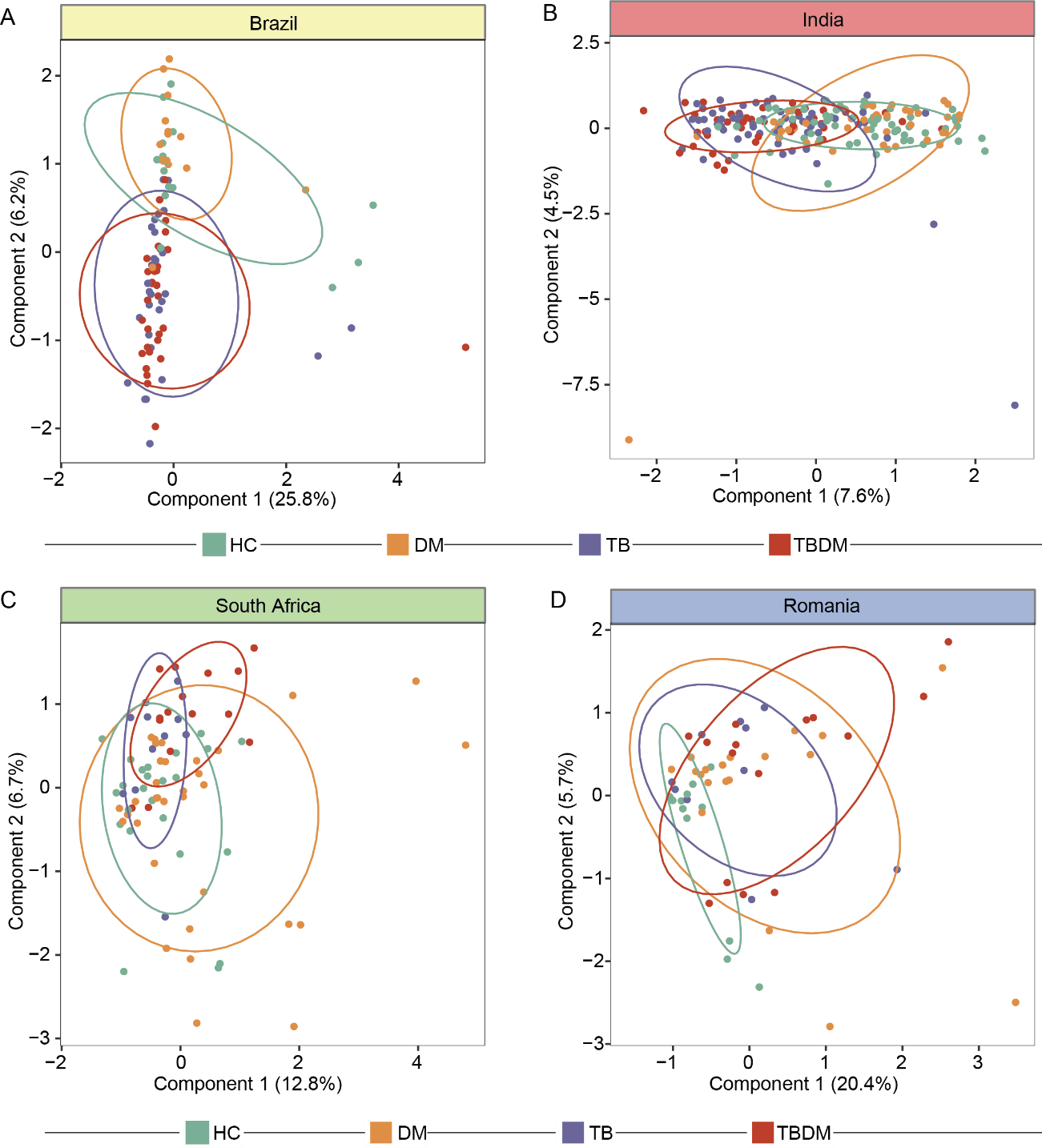


**Supplementary Fig. S2. Identifying TB participants using Differential Expression Genes (DEGs).** A principal component model was employed to test whether DEGs could cluster the patients in each clinical site, as indicated.


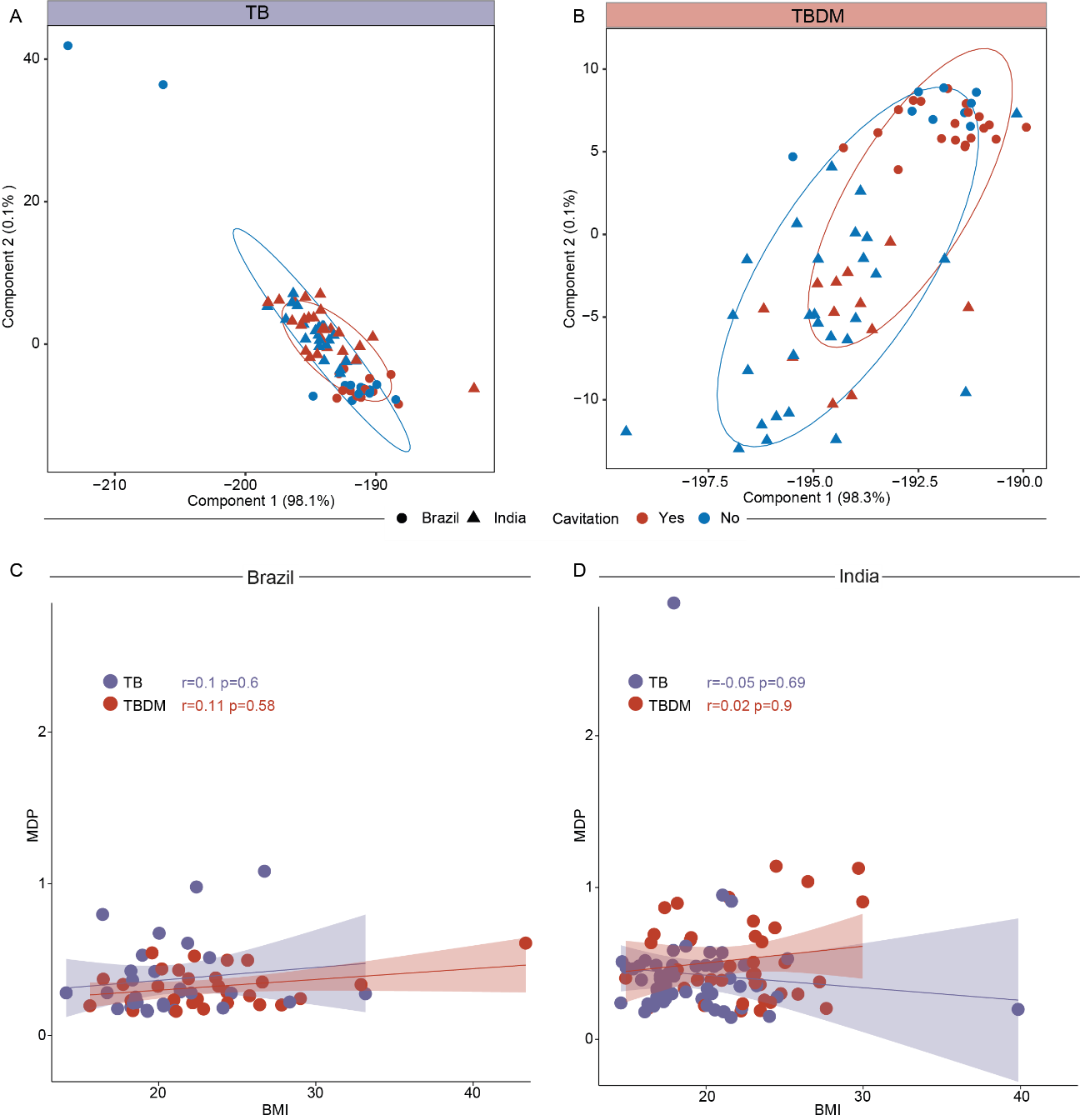


**Supplementary Fig. S3. Impact of clinical and epidemiological features in the gene expression variability. (A-B)** A principal component model was employed to test whether the presence or absence of cavitation could explain the differences in the gene expression profile between the countries. **(C-D)** A Spearman correlation analysis was used to evaluate if changes in BMI values are associated with the degree of the molecular degree of perturbation (MDP) in each clinical group.


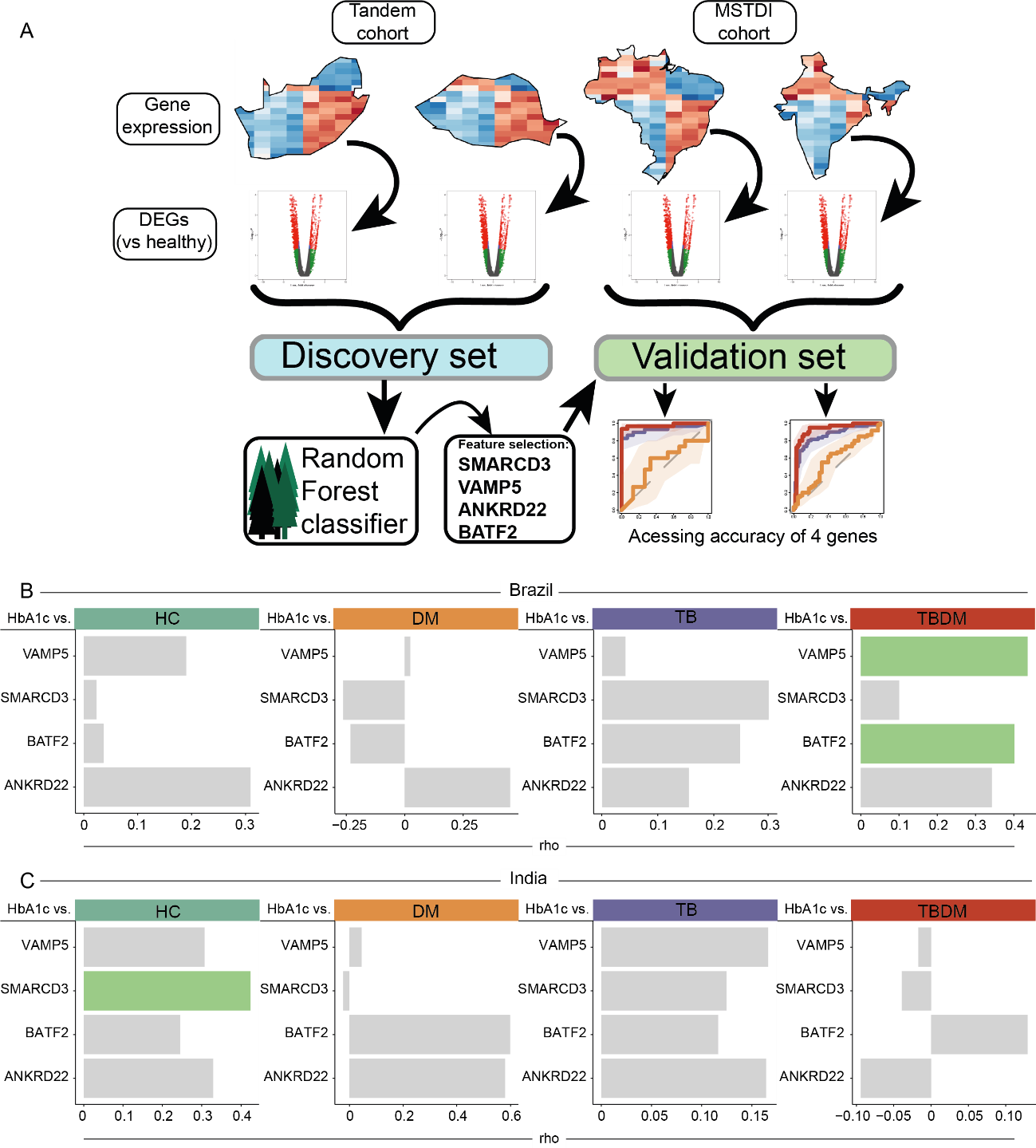


**Supplementary Fig. S4. Identifying the top genes expression associated with TBDM**. (A)DEGs values were inputted for a Random Forest model. South Africa and Romania sites were used as discovery set and the accuracy of the model was tested in the validation set composed of samples from the Brazil and India site. (B) A Spearman correlation analysis were performed between top genes expression and levels of HbA1c in each group from Brazil and India. Correlations with p-value < 0.05 were indicated as green bars.


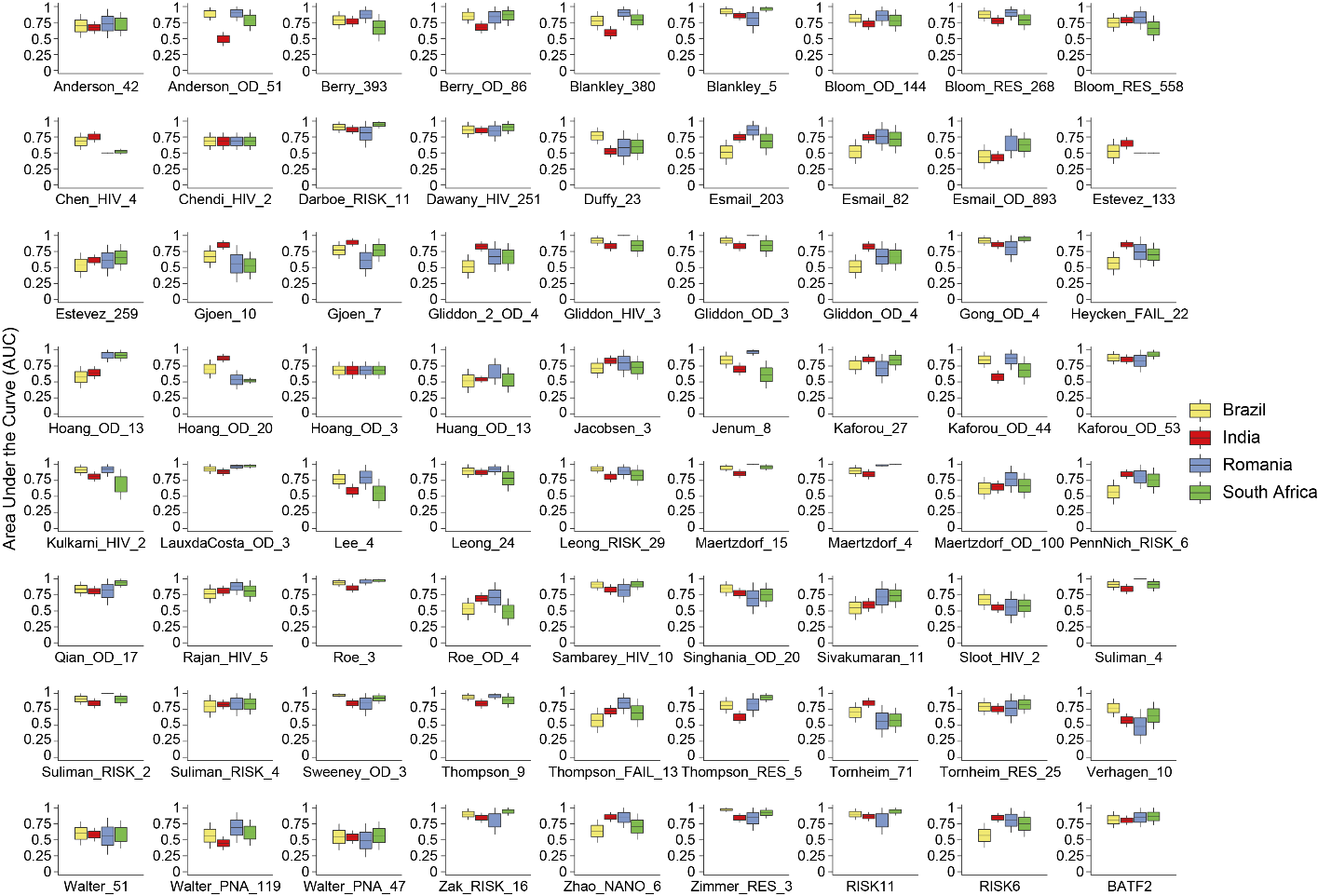


**Supplementary Fig. S5. Assessing performance of previously published gene biosignatures to identify TB cases in each country.** Receiver Operator Characteristics (ROC) analysis were performed to test the accuracy of previous reported signatures in our TB population. The y-axis shows the area under curve (AUC) values.


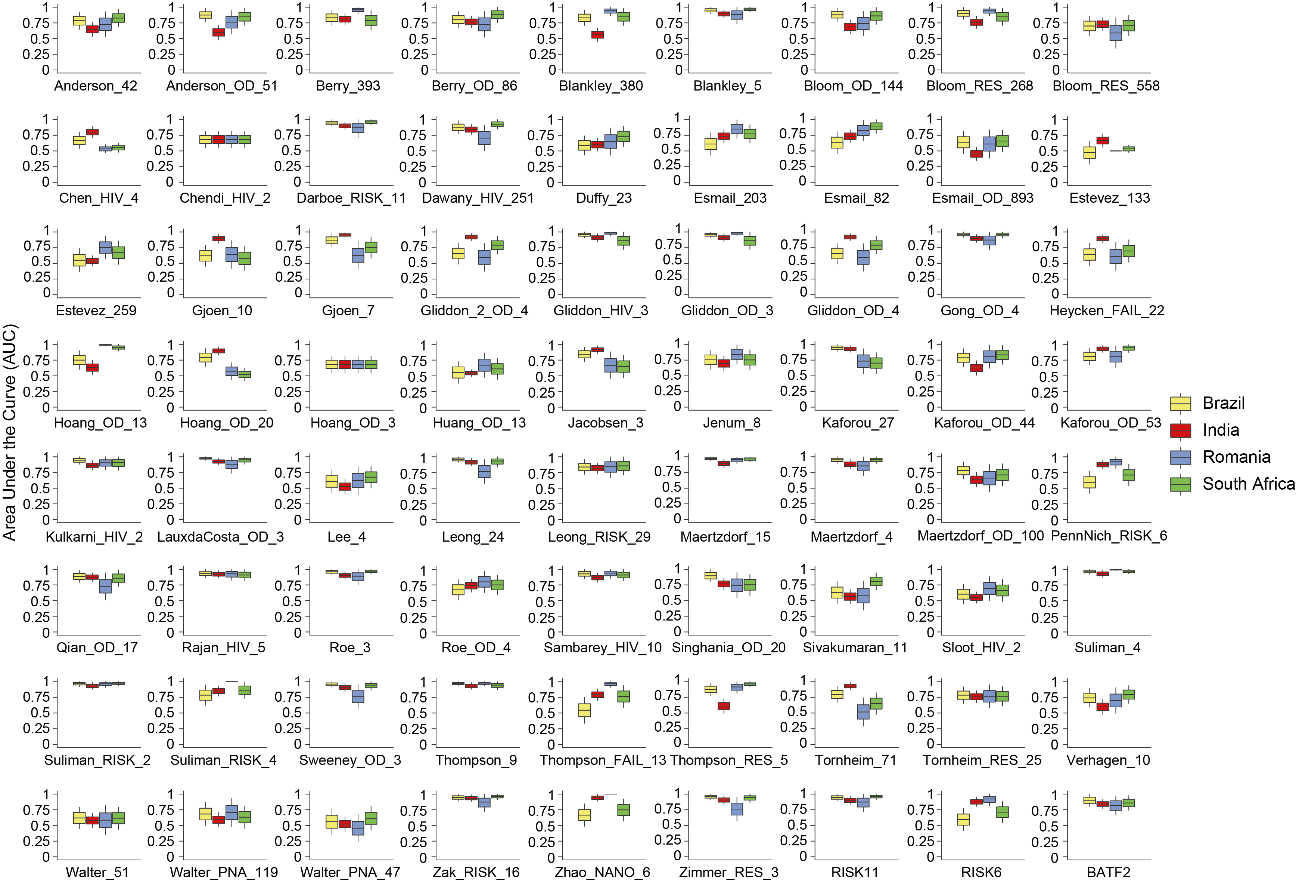


**Supplementary Fig. S6. Assessing performance of previously published gene biosignatures to identify TB-diabetes cases in each country.** Receiver Operator Characteristics (ROC) analysis were performed to test the accuracy of previous reported signatures in our TB population. The y-axis shows the area under curve (AUC) values.


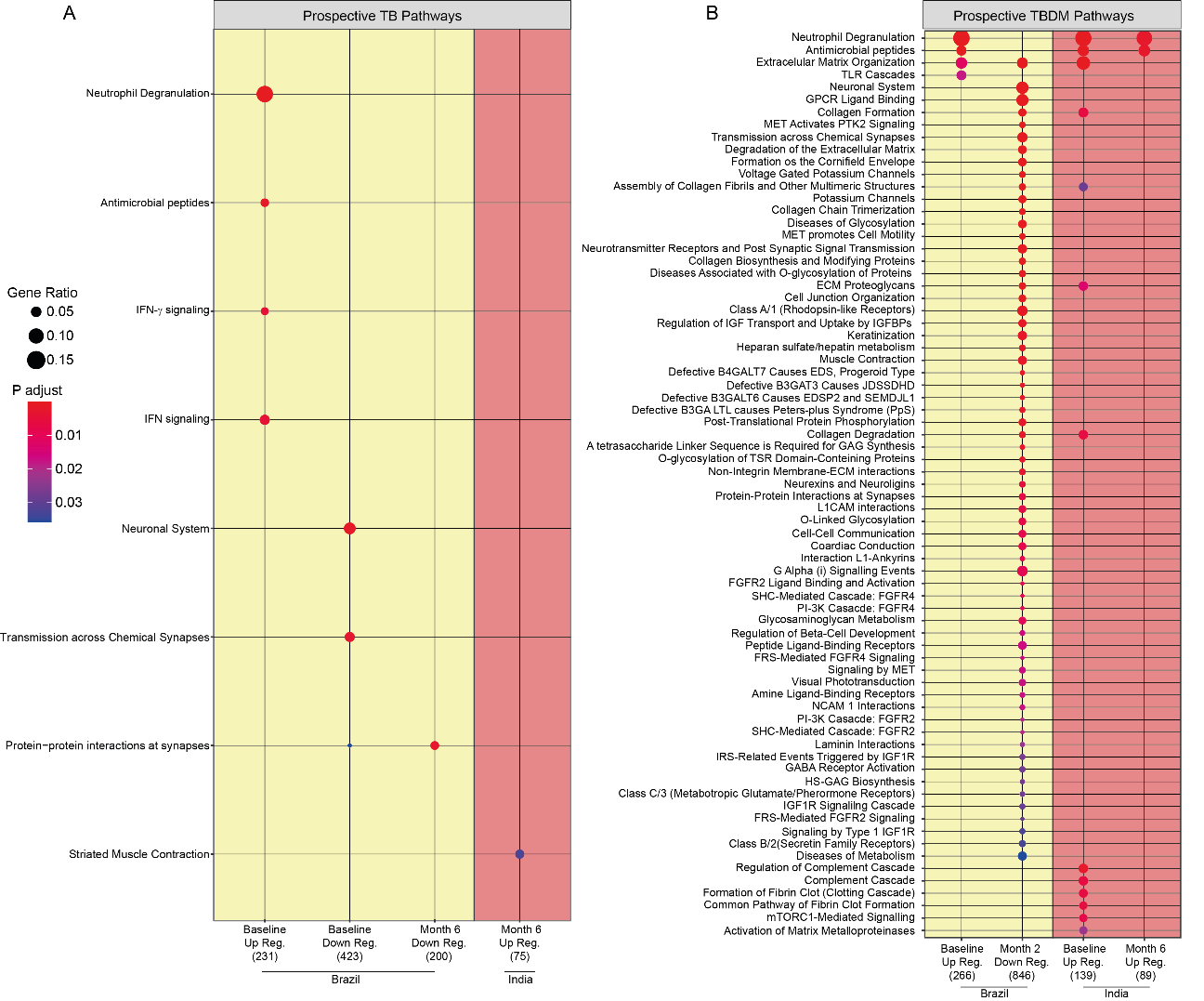


**Supplementary Fig. S7. Changes in pathways expression after started antitubercular therapy.** The colored spots indicate the enriched pathways identified from the DEGs of the comparisons of (A) TB and (B) TBDM in each time point with the HC. The sites are highlighted by colors: pathways from Brazi are colored yellow and India are colored red. The color gradient of spots corresponds to the FDR-corrected p-value and the size corresponds to the gene ratio.

**
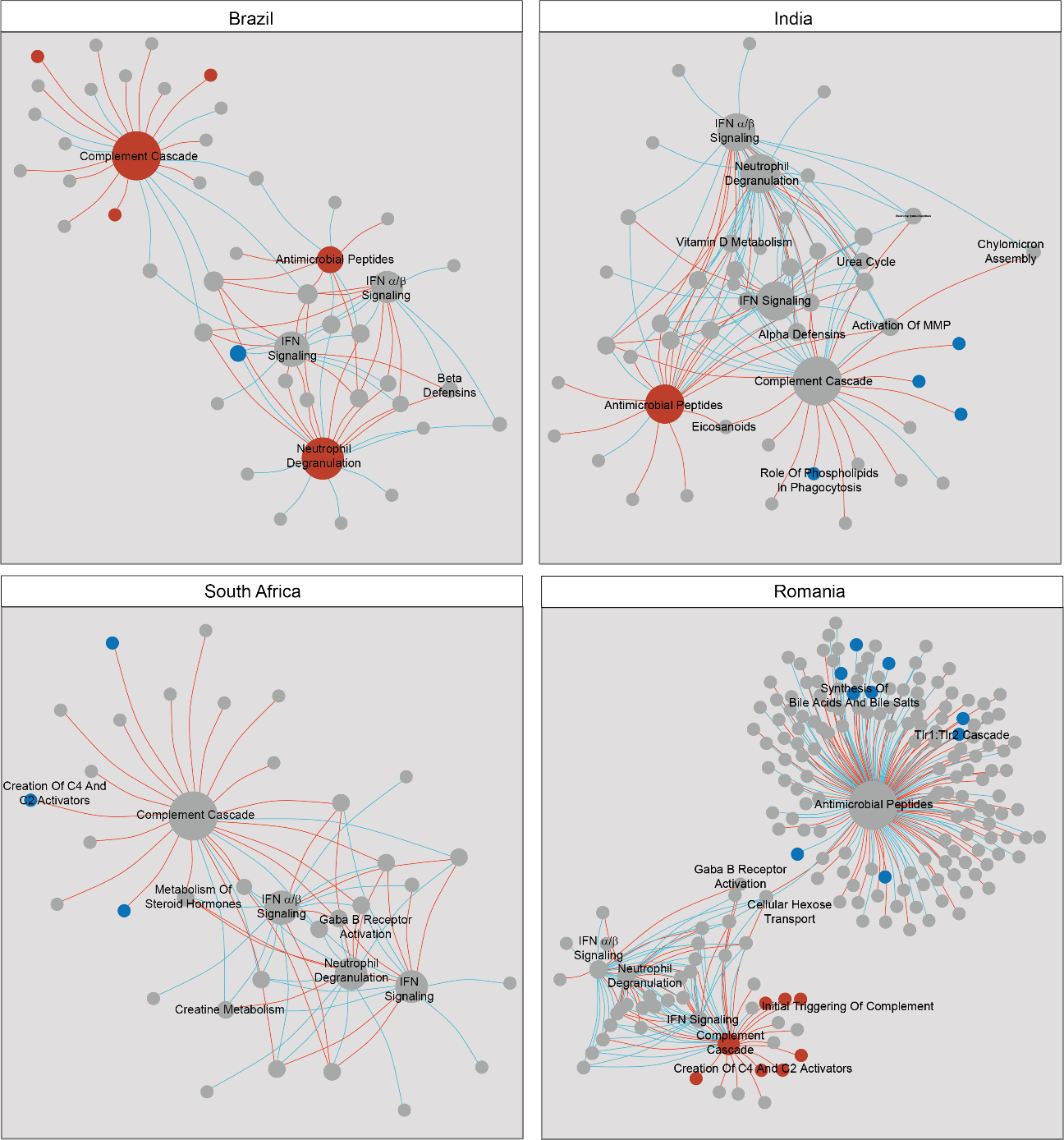
**

**Supplementary Fig. S8.** **Changes in the dynamicity of pathways across the clinical sites in TB**. A Spearman correlation analysis was performed using the pathways from TB participants in each clinical site, as indicated. Each node indicated a pathway, blue node infers downregulation when compared with control group, whereas red nodes upregulation. Red lines infer positive correlation and blue lines negative interaction.
